# Proteomic Analysis of Exosomes during Cardiogenic Differentiation of Human Pluripotent Stem Cells

**DOI:** 10.3390/cells10102622

**Published:** 2021-10-01

**Authors:** Preeti Ashok, Emmanuel S. Tzanakakis

**Affiliations:** 1 Chemical and Biological Engineering, Tufts University, Colby St., Medford, MA 02155, USA; Preeti.Ashok@tufts.edu; 2 Cell, Molecular and Developmental Biology, Tufts University, Harrison Ave., Boston, MA 02111, USA; 3 Tufts Medical Center, Clinical and Translational Science Institute, Washington St., Boston, MA 02111, USA

**Keywords:** human pluripotent stem cells, exosomes, differentiation, cardiomyocytes, stirred suspension culture, proteomics

## Abstract

Efforts to direct the specification of human pluripotent stem cells (hPSCs) to therapeutically important somatic cell types have focused on identifying proper combinations of soluble cues. Yet, whether exosomes, which mediate intercellular communication, play a role in the differentiation remains unexplored. We took a first step toward addressing this question by subjecting hPSCs to stage-wise specification toward cardiomyocytes (CMs) in scalable stirred-suspension cultures and collecting exosomes. Samples underwent liquid chromatography (LC)/mass spectrometry (MS) and subsequent proteomic analysis revealed over 300 unique proteins from four differentiation stages including proteins such as PPP2CA, AFM, MYH9, MYH10, TRA2B, CTNNA1, EHD1, ACTC1, LDHB, and GPC4, which are linked to cardiogenic commitment. There was a significant correlation of the protein composition of exosomes with the hPSC line and stage of commitment. Differentiating hPSCs treated with exosomes from hPSC-derived CMs displayed improved efficiency of CM formation compared to cells without exogenously added vesicles. Collectively, these results demonstrate that exosomes from hPSCs induced along the CM lineage contain proteins linked to the specification process with modulating effects and open avenues for enhancing the biomanufacturing of stem cell products for cardiac diseases.

## 1. Introduction

Exosomes mediate the intercellular transfer of information in the form of RNA and proteins in response to physiological processes under normal and diseased states. Exosomal RNA molecules have attracted attention for their roles in tissue regeneration including cardiac repair [1,2,3]. Yet, the protein content of cell- and body fluid-derived exosomes and its potential effects on cell physiology have remained surprisingly unexplored [4,5,6,7], especially when considering that microRNAs may be present within exosomes in suboptimal quantities to induce changes [8]. For instance, human-induced pluripotent stem cells (hiPSCs) overexpressing ALIX secrete exosomes with higher protein content and decelerate aging-related damage upon addition to skin fibroblasts [9]. Mesenchymal stem cells (MSCs) release exosomes containing proteins, which influence NF-κB signaling that promotes angiogenesis in HUVEC cells [10].

Various reports have centered specifically on exosomal proteins in the context of cardiac cell function and disease. The addition of MSC exosomes to ischemic myocardial cells induces an increase in ATP and NADH levels with concomitant reduction in oxidative stress [11]. The observed changes are attributed to glycolytic proteins such as GAPDH, PGK, PKM2, and ENO carried in exosomes. Similarly, exosomes secreted by cardiomyocytes (CMs) or cardiac progenitor cells (CPCs) ameliorate cardiac damage. The beneficial effect is ascribed to the exosome-born pregnancy-associated plasma protein-A (pappalysin; PAPPA) [12], which cleaves IGFBP-4 to release IGF-1 and reduce CM apoptosis. Exosomes from cardiac fibroblasts upregulate the renin-angiotensin system in cultured CMs in the presence of angiotensin II [13]. Proteomic analysis revealed the increased presence of MAPK and Akt pathway-controlling proteins in exosomes secreted by fibroblasts treated with angiotensin. Conversely, extracellular vesicles from hiPSC-derived CMs added to bovine aortic endothelial cells result in significant gains in tube formation, wound closure, and cell proliferation [14].

Despite the documented capacity of exosomal proteins for cardiac repair, there are no reports to date on the potential role of exosome-carried proteins on the commitment of human pluripotent stem cells (hPSCs) to CMs. Embryonic cardiopoiesis originates at the mesoderm [15], and it is influenced by factors secreted by the neural ectodermal [16] and endodermal [17] layers. It is plausible that exosome delivery of such factors is a transport modality operating during embryonic differentiation. This raises questions as to whether exosome-carried factors contribute to the differentiation process in vivo and in cultured hPSCs.

As a first step towards addressing this issue, we set out to characterize the protein content of exosomes at various stages of the cardiogenic specification of hPSCs. Human embryonic stem cells (hESCs) and hiPSCs were coaxed to mesoderm-oriented primitive streak, cardiac mesoderm, and cardiac muscle cells [18]. Exosomes released at each of these differentiation stages were harvested and subjected to proteomic analysis. Changes were observed in the protein cargo of exosomes as the cells transitioned toward CMs. An assortment of proteins contained in exosomes were documented including several moieties modulating signaling—such as TGFβ and canonical Wnt—linked to the specification of hPSCs to CMs. In addition, we report that exposure of hPSC-derived cardiac mesoderm cells to exosomes from hPSC-derived CMs induced an increase in the fraction of CM progeny.

## 2. Materials and Methods

### 2.1. Human Pluripotent Stem Cell Culture

H9 hESCs (WiCell, Madison, WI, USA, passages 35–50) and B12-3 hiPSCs (Harvard Stem Cell Institute, Cambridge, MA, USA, passages 20–35) were cultured on dishes coated with Matrigel (Corning Inc., Corning, NY, USA). Cells were passaged as colonies every 5–7 days using collagenase Type IV (ThermoFisher Scientific, Waltham, MA, USA) enzyme. For dissociation into single cells, colonies were incubated with Y-27632 (Enzo Life Sciences, Farmingdale, NY, USA) Rho-associated protein kinase (ROCK) inhibitor for 1 h before treatment with Accutase (ThermoFisher, Waltham, MA, USA). Single dispersed cells were seeded in the spinner flask as we reported [19]. The number and viability of cells were determined by staining culture samples with the Trypan Blue dye (ThermoFisher, Waltham, MA, USA) and using a hemocytometer or the TC20 cell counter (Bio-Rad, Hercules, CA, USA).

### 2.2. Cardiac Differentiation

Xeno-free differentiation of hPSCs toward cardiomyocytes was carried out as we described recently [18]. Briefly, 5 × 10^6^ cells were seeded as single cells in spinner flasks (Corning Inc., Corning, NY, USA) with 50 mL StemMACS iPS-Brew XF culture medium (Miltenyi Biotech, Auburn, CA, USA). After 5 days of hPSC expansion as aggregates, the medium was replaced with xeno-free differentiation medium (DM) supplemented with 200 ng/mL WNT3A and 30 ng/mL BMP4. Medium was changed after 24 h. Forty-eight hours later, DM with 10 µM KY02111 (R&D Systems, Minneapolis, MN, USA) Wnt inhibitor was added and replaced daily for 6 days. Cells were then cultured in DM until day 13 with daily medium exchange.

### 2.3. Exosome Isolation and Characterization

**Figure 1:**
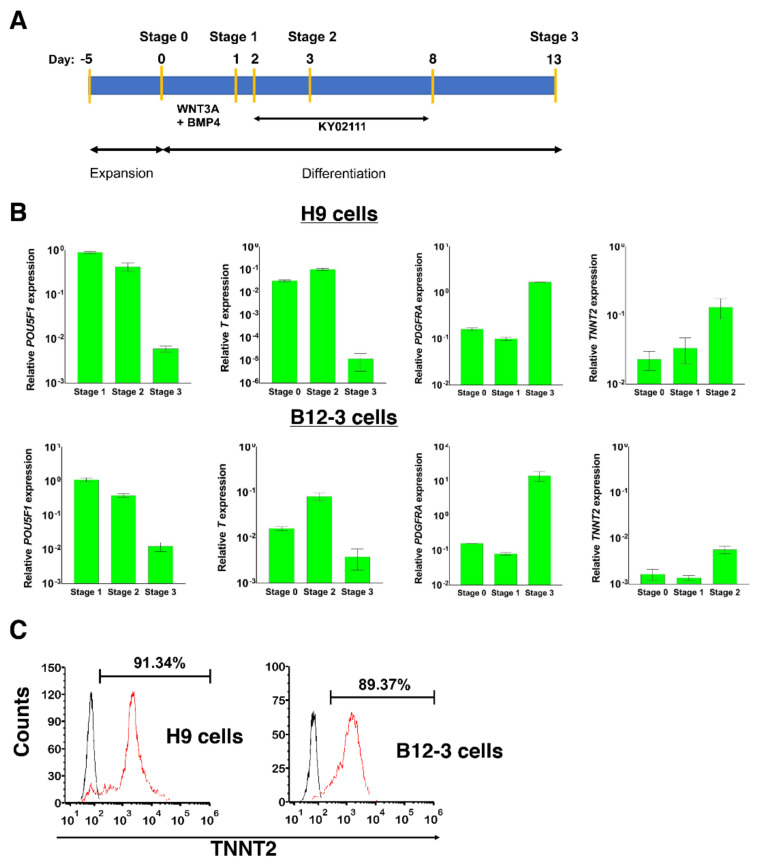
Human PSC differentiation toward CMs. (**A**) Schematic of the protocol implemented for cardiogenic specification of hPSCs. (**B**) Relative gene expression during differentiation assessed by qPCR. Results are shown as mean ± SD (*n* = 3) relative to the expression at a specific stage: *POU5F1*-stage 0 (S0), *T*-S1, *PDGFRA*-S2, *TNNT2*-S3. (**C**) Expression of TNNT2 in differentiated hPSCs on day 13 (stage 3). Results from representative flow cytometry runs are shown. Curves: black-Isotype, red-TNNT2.

Exosomes were isolated following a standard protocol [20] with modifications. Stem cells were expanded for 5 days in spinner flasks and then differentiated into cardiomyocytes. Medium was collected on days 3, 4, and 5 (and pooled) of expansion, and days 1, 3, and 11–13 of differentiation for exosome isolation (Figure 1A). The medium was centrifuged at 1000× *g* for 15 min at 4 °C to eliminate cells, and again at 16,500× *g* for 20 min at 4 °C to remove larger vesicles, followed by filtration through a 0.22 μm filter. The filtrate was ultracentrifuged at 100,000× *g* (70 Ti rotor, Beckman Coulter, Danvers, MA, USA) at 4 °C for 120 min. The supernatant was removed but 1 mL for resuspending the pellet of exosomes. Suspensions corresponding to the same culture were combined into a single tube, washed with phosphate buffer saline (PBS), and an identical round of centrifugation was performed. The supernatant was discarded, and the pellet was resuspended in 100 μL PBS.

A sucrose gradient was performed by adapting a published protocol [21]. Briefly, a series of sucrose (Sigma-Aldrich, St. Louis, MO, USA) solutions (10%, 16%, 22%, 28%, 34%, 40%, 46%, 52%, 58%, 64%, 70%, and 90% (*w/v*)) were made in PBS. The exosome pellet was suspended into 1 mL of the 90% sucrose stock in an ultracentrifuge tube. To form the sucrose gradient, the rest of the solutions were overlayed on top of the exosome suspension at a volume of 1 mL each. Ultracentrifugation (Optima XE (Beckman Coulter, Indianapolis, IN, USA); SW32 swinging bucket rotor) was performed at 100,000× *g* for 16 h. Six fractions of 2 mL each were collected without disturbing the gradient. The fractions were washed with PBS and analyzed for exosomal markers by western blotting (see relevant section).

### 2.4. Size and Morphological Analysis

Extracted exosomes were diluted to 0.1 μg/μL using Dulbecco’s PBS (DPBS) and transferred to a cuvette (100 μL). Size analysis was done using dynamic light scattering (DLS; Nanobrook ZetaPals analyzer, Brookhaven Instruments, Holtsville, NY, USA) with the following parameter values: Temperature = 25 °C, number of runs = 3, and run duration = 120 s. Polydispersity was calculated and ensured to be less than 0.3. The size or homogeneity of exosomes was recognized through the diameter or the % number, respectively.

TEM analysis of harvested exosomes was carried out at the Whitehead Institute (Cambridge, MA, USA).

### 2.5. RNA Extraction, RT-PCR and Quantitative PCR Analysis

Total cellular RNA was extracted with Trizol (ThermoFisher, Waltham, MA, USA) according to the manufacturer’s instructions. Reverse transcription was performed at 70 °C for 5 min and 42 °C for 60 min with 1 µg total RNA using ImProm-II reverse transcriptase (Promega, Madison, WI, USA) and 250 ng oligo(dT)_12–18_ primers (ThermoFisher, Waltham, MA, USA). The resulting complementary DNA (cDNA) was analyzed with a StepOne Plus thermocycler (Applied Biosystems, Foster City, CA, USA) by quantitative PCR (qPCR) for 40 cycles and 58–60 °C annealing temperature depending on each primer set. MicroRNA (miRNA) cDNA was synthesized in two steps (qScript microRNA cDNA synthesis kit, QuantaBio, Beverly, MA, USA): adding the polyA tail by cycling 1 μg RNA with polyA primers and polymerase at 37 °C for 60 min and at 70 °C for 5 min. Reverse transcription was done incubating the resultant solution with dNTPs, reverse transcriptase, and appropriate buffers at 42 °C for 20 min and 85 °C for 5 min. Primer sequences are shown in Table S1 including a universal primer for miRNA qPCR. Analysis was performed based on the ΔΔC_T_ method [22] with *ACTB* as the housekeeping gene for mRNA, and *RNU6* for miRNA.

Expression for each marker gene was quantified relative to the expression level at the marker’s specific stage, i.e., stage 0 (undifferentiated hPSCs) for *POU5F1*, stage 1 for *T*, stage 2 for *PDGFRA*, and stage 3 for *TNNT2*.

### 2.6. Western Blot Analysis

Total protein was isolated using lysis buffer containing Tris-HCl (50 mM, pH 8), NaCl (150 mM), NP40 (1%), sodium dodecyl sulfate (SDS) (0.1%), sodium deoxycholate (1%), protease inhibitor cocktail including phenylmethanesulfonyl fluoride (PMSF) (Sigma-Aldrich, Burlington, MA, USA), and phosphatase inhibitors (1 mM sodium fluoride, 5 mM sodium pyrophosphate, 5 mM sodium orthovanadate). Protein concentration was determined via the Bradford method (Pierce Biotechnology, Waltham, MA, USA). Protein lysates were boiled at 95 °C in Laemmli sample buffer (Bio-Rad, Hercules, CA, USA) and about 20 µg were loaded in a polyacrylamide gel along with a biotinylated protein ladder (Cell Signaling Technology, Beverly, MA, USA) as described [23]. After protein transfer, the polyvinylidene difluoride (PVDF) membranes were blocked with 5% non-fat dry milk in Tris-buffered saline containing 0.1% Tween 20 (TBST) and primary antibodies against CD63 and GRP94 (Santa Cruz Biotechnology, Dallas, TX, USA), LAMP1 and LAMP2 (Abcam, Cambridge, MA, USA), in blocking buffer for overnight incubation on a rocker at 4 °C. Antibody (Sigma) was used also for detection of ACTB serving as a loading control. Membranes were washed four times (10 min each) with TBST and incubated for 1 h at room temperature with corresponding horseradish peroxidase (HRP)-linked secondary antibodies (Jackson ImmunoResearch Laboratories, West Grove, PA, USA). After further washing with TBST, chemiluminescence reagent (Pierce) was added and the membranes were scanned with a C-DiGit blot scanner (LI-COR Biotechnology, Lincoln, NE, USA).

### 2.7. Proteomic Analysis

Sample protein was prepared using Filter-Aided Sample Preparation (FASP) [24]. Exosome proteins were retained in 10 kDa filters (MilliporeSigma, Burlington, MA, USA), enabling salt-laden solvents to be separated by centrifugation. Reduction, alkylation, and digestion using trypsin were all done in the filter. After 12 h of digestion at 37 °C, peptides were obtained by centrifugation and collection of the effluent with minimal salt concentration.

The LC system used an Ascentis Express Peptide ES-C18 column (Sigma Aldrich, Burlington, MA, USA) with a 2 h gradient run at 100 μL/min, using water (0.1% Formic Acid) and acetonitrile (0.1% Formic Acid) as mobile phases. An IDA (information-dependent analysis) method of data acquisition was used with TOF (time of flight) MS and MS/MS to determine the proteins present in the exosome samples [25]. IDA scans for 25 ions/cycle were performed, with dependent scans induced for precursor ions with *m/z* = 300–1250 Da and +2 to +5 charges. Ions within 4 Da were excluded and a mass tolerance of 50 Da was included. The Protein Pilot software (ver. 5.1, AB SCIEX, Framingham, MA, USA) was used to identify the proteins. A comparison of the peak intensities of the first three peptides was performed and used for comparison among of datasets. This serves as a relative quantitation of the respective proteins. All data were normalized to exosome mass, and the amount of albumin present in each sample. Albumin was chosen as an external control because it was the only detected protein that is added to media.

### 2.8. Addition of Exosomes to Differentiating Cells

Exosomes were isolated from media harvested from cultures on days 12, 13, and 14 of differentiation of B12-3 into CMs (stage 3) in spinner flasks. Fifty µg of exosomes were added to each confluent well of a 12-well plate containing differentiating hPSCs at stage 2 (day 3). The addition was staggered with half of the exosomes added on day 3 and the rest on day 5. As controls, differentiation was performed (i) without the addition of exosomes (no treatment), or (ii) the same amount of HEK 293 cell-derived exosomes was added at the same time points as above. Cell samples were collected on day 8 for analysis.

### 2.9. Flow Cytometry

Flow cytometry was performed by fixing cells in 4% formaldehyde in PBS for 15 min. Samples were washed three times, and permeabilized using Triton-X (Acros Organics, Fair Lawn, NJ, USA). After further washing, samples were blocked with 3% normal donkey serum (NDS) (Jackson ImmunoResearch, West Grove, PA, USA). Primary mouse antibody for MYH1E (MF20; Developmental Studies Hybridoma Bank, Iowa City, IA, USA) and secondary donkey anti-mouse Dylight 649 antibody (Jackson ImmunoResearch) were suspended in 1% NDS before incubation at room temperature for 1 h. Flow cytometry was performed with the Attune Nxt flow cytometer (ThermoFisher, Waltham, MA, USA) and analysis was carried out with the FCS express software (v. 7.0, De Novo Software, Glendale, CA, USA). Samples were analyzed against isotype controls.

### 2.10. Statistical Analysis and Experimental Design 

Data are expressed as mean ± standard deviation (SD) unless stated otherwise. Student t-tests were performed with values of *p* < 0.05 considered as significant. The ClueGO suite was used to perform gene ontology enrichment and relevant data visualization. PCA plots were created and Pearson correlation coefficient (PCC) values for dataset pairs were calculated using the JMP Pro software (SAS, Cary, NC, USA). The PCC was calculated based on the levels of proteins detected in both pair sets. Permutational multivariate analysis of variance (PERMANOVA, Mölndal, Sweden) was performed using R (v. 4.0).

## 3. Results

### 3.1. Human Pluripotent Stem Cell-Secreted Exosome Collection and Characterization

Human PSCs were expanded and differentiated as aggregates in spinner flask cultures in a stage-wise fashion [18] (Figure 1A): Stage 0—hPSCs expressing POU5F1 (OCT4); Stage 1—mesodermal cells at 24 h of differentiation expressing Brachyury (T); Stage 2—cardiac mesoderm cells emerging after 72 h of differentiation (24 h of Wnt suppression) expressing PDGFRA and NKX2.5; Stage 3—CM-like cells expressing cardiac Troponin T (TNNT2). The rise and fall in expression of the above markers at various stages were monitored by qPCR (Figure 1B). Beating aggregates were observed by day 9 with 91 ± 0.71% (*n* = 3) of H9 cells and 86.67 ± 3.71% (*n* = 3) of B12-3 hPSCs being TNNT2^+^ by day 12 (Figure 1C).

**Figure 2:**
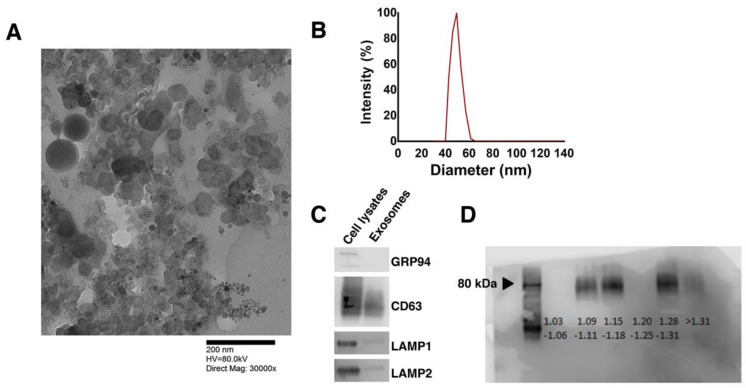
Morphological and biochemical characterization of hPSC-derived exosomes. (**A**) TEM image and (**B**) size distribution (DLS analysis) of exosomes released by hPSCs. (**C**) Western blotting of cell lysates and exosomes for GRP94 (100 kDa), CD63 (35–75 kDa), LAMP1 and LAMP2 (90–120 kDa). See whole blots in Figure S1. (**D**) Western blot detection of CD63 performed on various layers of a sucrose density gradient. Each lane corresponds to a density range (g/mL) as indicated.

Between 20–40 μg exosomes (based on total protein) were isolated routinely from 200–300 mL media over 6 days of hPSC cultivation. Media were harvested over the final three days of expansion, centrifuged at 300× *g* and 16,000× *g* to remove cell debris and larger vesicles, filtered, and stored at −80 °C until exosome preparation. Isolated exosomes appeared spherical in transmission electron micrographs (Figure 2A) with a distribution of sizes between 32–66 nm and averaging 50 nm (Figure 2B). The presence of the exosomal markers CD63, LAMP1, and LAMP2 was confirmed by western blotting (Figure 2C). It should be noted that these proteins are absent from the plasma membrane indicating the cytoplasmic origin of the vesicles. GRP94, which resides in the endoplasmic reticulum, was not detected suggesting that the isolates did not contain impurities due to cell lysis. Moreover, exosomes appeared in the 1.15–1.18 g/mL density range by sucrose gradient analysis [26] (Figure 2D). The fractions above 1.25 g/mL represent agglomerated proteins and RNA (lanes 5–6), while Golgi vesicles appear in the 1.05–1.12 g/mL range. Exosomes reportedly are observed in fractions 3–5 [26] as our gradient analysis indicates.

Lastly, the addition of 100 µg of exosomes released by H9 hPSCs to HEK293 cells resulted in the upregulation of microRNA-302C and -302A, which are enriched in hPSCs [27] (Figure S2). The data show that the isolated vesicles exhibit characteristics which are typical of exosomes with cargo inducing changes in the expression of recipient cells.

### 3.2. Proteomic Cargo of Exosomes during Cardiogenic hPSC Differentiation

Exosomes were harvested from cultures at each stage of differentiation. Exosomes from undifferentiated (stage 0) and committed hPSCs (stages 1–3) were subjected to proteomic analysis. Duplicate samples collected from the H9 and B12-3 hPSCs resulted in eight different dataset groups. Between 100–300 proteins were detected per sample with 100–183 proteins shared among duplicates. A list of 337 unique proteins was compiled (Table S2). Two keratin isoforms and trypsin due to sample preparation were excluded from our analysis. The proteins were verified against Exocarta [28], a curated database of exosomal proteins. Of the proteins shared among the exosome groups, 39 were strictly nuclear. Some of the detected proteins (FAU, LGI1, CRABP1, HNRNPA1, RPL36AL, SRSF12, SLC7A3) are present in multiple subcellular locations including the nucleus, and cytoplasm and/or extracellular space. Exosomes under every condition had 18 proteins in common (Table S3) including metabolic, exosomal, and proliferation-related proteins.

**Figure 3:**
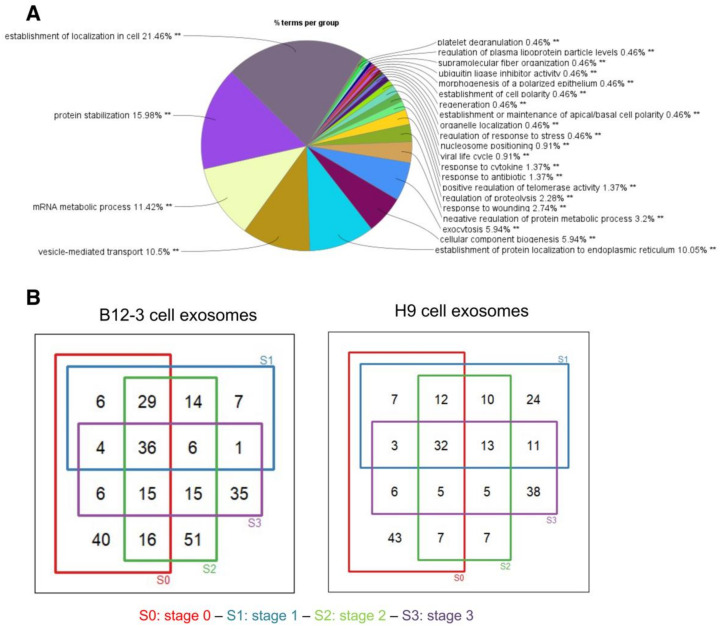
Gene ontology (GO) and quantification of the number of proteins shared among exosomes harvested from various stages of cardiogenic differentiation. (**A**) GO analysis of all proteins found in exosomes from H9 and B12-3 hPSCs coaxed toward CMs. (**B**) Venn diagrams of exosomal proteins at different stages (S0 to S3) of the cardiogenic commitment of B12-3 and H9 hPSCs.

A gene ontology (GO) analysis of all 330 identified proteins (Figure 3A) revealed that approximately 37% of them are ribosomal involved in protein translation, signal recognition particle (SRP)-dependent localization, and co-translation. Another 18% of the proteins relates to protein stabilization including several extracellular proteins, whereas a fraction (~13%) of the identified proteins is linked to exosome production, exocytosis, vesicle-mediated transport, and apoptotic signaling. The remaining proteins mediate metabolic processes, signaling and reorganization of cellular components.

For proteins appearing in both duplicate samples of each dataset, Venn diagrams were constructed to illustrate the number of overlapping proteins at various stages in both hPSC lines (Figure 3B). The common proteins in B12-3 cells (36) consisted of exosomal (HSPA8, ANXA2, HSP90AB1), ribosomal/translational, metabolic (GAPDH, PKM, FASN, ENO1), histone, and cytoskeletal proteins. Similarly, the common proteins of the H9 line vesicles (31) consisted of exosomal (HSPA8, HSP90AB1), ribosomal/translational, metabolic (ENO1, PKM, GAPDH), cytoskeletal, and histone proteins.

**Figure 4:**
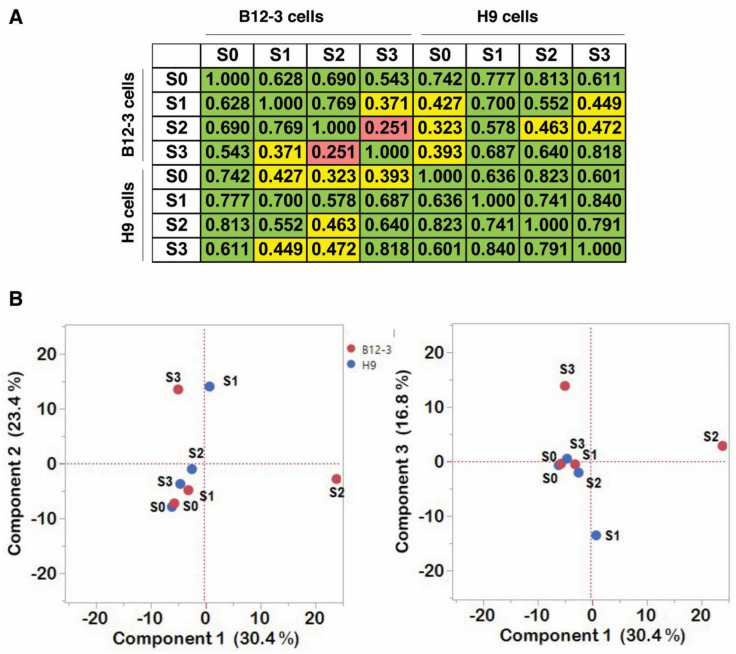
Correlational and dimensional reduction analyses of proteomic content of isolated exosomes. (**A**) Value of the PCC upon pairwise comparison of exosomal samples from different stages of differentiation. (**B**) PCA of all available datasets in this study. The first three PCs are shown. Numbers in parentheses show the variance attributed to the respective component. The hPSC line from which the exosomes are derived is shown by color: B12-3-red, H9-blue. Stages are indicated by ‘S’ followed by a numeral, e.g., stage 2 is S2.

The Pearson correlation coefficients (PCC) were calculated for different datasets to determine whether exosomal protein content changes are correlative with differentiation stage and hPSC line (Figure 4A). Based on the PCC values exosomal protein content from either hPSC lines is strongly correlative (PCC > 0.4) for most differentiation stages except for the B12-3 cells between S1 and S3 (PCC = 0.371), and S2 and S3 (PCC = 0.251). Exosomal cargo from undifferentiated H9 cells displayed moderate correlation with that from differentiating B12-3 cells in S2 (PCC = 0.323).

We performed PCA for the entire dataset (Figure 4B). The highest variance (PC1) demonstrated that B12-3 S2 (BS2) was the only set that exhibited marked separation from the rest of the sets. This was likely due to the presence of the greatest number of unique proteins present in the set. Especially pronounced was the split between exosomes from S1 and S3 of the two cell lines along the second highest variance (PC2). Interestingly, the plot of PC1 vs. PC3 shows the same divide for these stages. The three PCs explained over 70% of the data variance. PERMANOVA was performed to indicate the uniqueness of the B12-3 S2 when compared to the other datasets, yielding a *p* value of 0.001. It should be noted that the variation in exosomal protein content and quantity is expected. For instance, S1 (mesoderm) is characterized by a high proportion of T^+^ cells being present for only ~24 h. Moreover, cell populations at various stages are heterogeneous. For instance, S2 ensembles contain ~20% NKX2.5^+^ cells and smaller fractions of KDR^+^ and PDGFRA^+^ cells [18] depending on the hPSC line.

**Figure 5:**
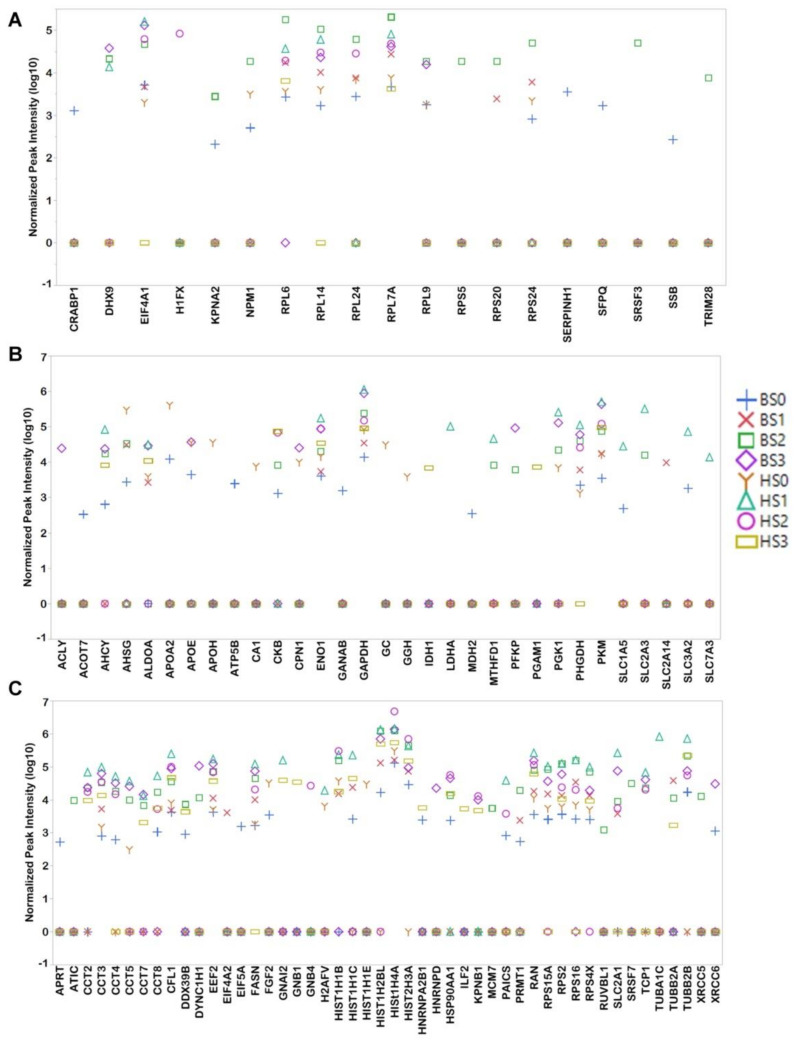
Relative abundance of proteins in exosomes from hPSCs undergoing differentiation toward CMs. Scatterplots of (**A**) pluripotency-, (**B**) metabolic-, and (**C**) proliferation-related proteins. The points are denoted by the hPSC line (B: B12-3, H: H9) followed by the stage of differentiation. For instance, HS2 corresponds to differentiation stage 2 for exosomal samples from H9 cells. Zero peak intensity means that a protein was not detected in a set.

Proteins linked to pluripotency (Figure 5A), metabolism (Figure 5B) and proliferation (Figure 5C) were visualized using scatter plots. Pluripotency-related proteins (19) account for 5.6% of the total proteins detected in the vesicles. Along with exosomes from S0, those from S1 and S2 contained pluripotency-related proteins (Figure 5A) with S2 exosomes from B12-3 cells exhibiting a higher number (15 proteins). Ribosomal/translational proteins (7) are present in exosomes at multiple stages with their highest content at S1 or S2 exosomes. Cytokine/transcriptional proteins (9) are present at a single stage or at the most two stages per line. The pluripotency-related proteins do not include prominent markers such as NANOG or POU5F1, but downstream or helper proteins (e.g., NPM1, KPNA2) which enable self-renewal and maintenance of pluripotency. These proteins are present at S1 or S2 of differentiation, indicating their possible intercellular discharge and dissemination via exosomes.

Metabolic proteins (31) (Figure 5B) are displayed differently in exosomes depending on the cell line. Among glycolysis proteins (GAPDH, ENO1, PKM, PFKP, PGK1) in the B12-3 cells, GAPDH, ENO1, and PGK1 are the most abundant at S3. The content of these proteins is the highest at S1 for H9 cells. ALDOA and PKM, which are glycolytic enzymes that are most abundantly expressed in muscle cells, have the same exosomal appearance pattern as the other glycolytic proteins. Along the same vein, the oxidative phosphorylation-catalyzing protein PHGDH is detected at maximum level in S3 for B12-3 and S1 for H9 cells. The fatty acid (FA) transporters APOE, APOA2, and APOH are present in exosomes from S0 or S0 and S3 of one or both hPSC lines. Fatty acids are required for cell proliferation potentially explaining the presence of the above-mentioned transporters in hPSC exosomes. Moreover, CMs metabolize FAs, in line with the detection of the FA transporters in S3. The same pattern was noticed for FASN, which is a protein with roles in metabolism and cell proliferation.

Cell proliferation-related proteins (48) (Figure 5C) were present in exosomes at all stages, including in S3 possibly due to the presence of immature CMs. It should be noted that the CMs derived here have functionality and characteristic markers similar to those of CMs derived by other differentiation protocols [29,30]. The proteins present in multiple datasets consisted of histones (6), T-complex proteins (7), and elongation factors (1). While the detected level of most of the above proteins was the highest in exosomes from S1 and S2, some such as HIST1H4A in the B12-3 cells were enriched in S3. G-proteins (GNAI2, GNB1, GNB4) were present in only H9 cell exosomes with GNB1 and GNB4 found only at S3.

### 3.3. Exosomes Contain Proteins which Influence hPSC Differentiation

**Figure 6:**
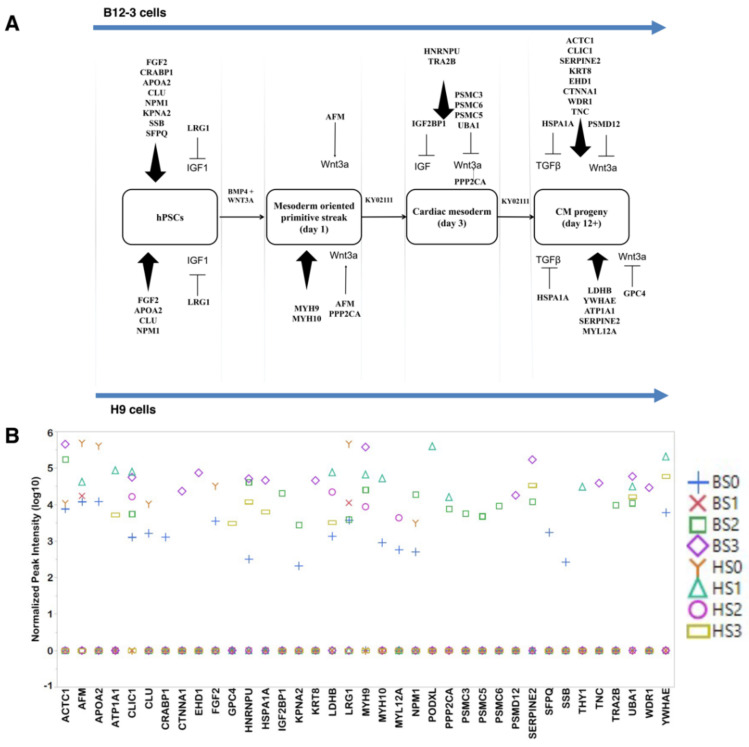
Detected exosomal proteins with potential roles in hPSC specification toward CMs. (**A**) Proteins that influence differentiation, and (**B**) their relative abundance depicted by a scatter plot.

Exosomes from one or more stages contained proteins known to influence differentiation (Figure 6A), signaling, cellular/ECM reorganization, or being considered differentiation markers. Stage 1 is induced through activation of canonical Wnt signaling, and respective exosomes contained the Wnt effector proteins AFM and PPP2CA in H9 cell cultures. Only AFM was present in B12-3 cell exosomes. Stage 2 exosomes of the B12-3 hPSCs also contained proteosome complex proteins (PSMC3, PSMC5, PSMC6), which degrade β-catenin, possibly to extinguish the canonical Wnt activation of the previous stage.

Mesodermal markers (MYH9, MYH10) were prevalent in S1 exosomes from H9 hPSCs, while cardiac mesodermal effectors (HNRNPU, TRA2B) were detected in S2 exosomes of B12-3 cells. More cardiac cell markers were present (12 vs. 4; Figure 6A,B) in exosomes from B12-3 than H9 cells in S3. CTNNA1, a mature cardiac cell marker was detected in B12-3-derived CM exosomes. Interestingly, cardiac proteins such as ACTC1 and MYL12A were also found in S0 exosomes.

Apart from cardiac markers, metabolic proteins relevant to cardiac tissue (H9 cells: ATP1A1, LDHB) and those involved in contractile activity (B12-3 cells: C1IC1, WDR1, EHD1; H9 cells: YWHAE) were identified.

### 3.4. Effect of Exosomes on Differentiation

Most of the exosomal proteins are detected only at a single stage. This timing suggests a potential role of the proteomic cargo of the exosomes in the differentiation process. Yet, we do not know if proteins are present in sufficient amounts to induce or tune a specification effect. To this end, we determined the extent of exosomal influence on differentiation by adding exosomes from S3 to S2 differentiating B12-3 cells. Exogenous exosome treatment was staggered with equal quantities being added at days 3 (S2) and 5. We performed the experiment on the B12-3 line due to the presence of a relatively high number of cardiogenic proteins in S3 exosomes. The H9 line contained several proliferation-promoting proteins (as in S1) and fewer cardiogenic proteins.

**Figure 7:**
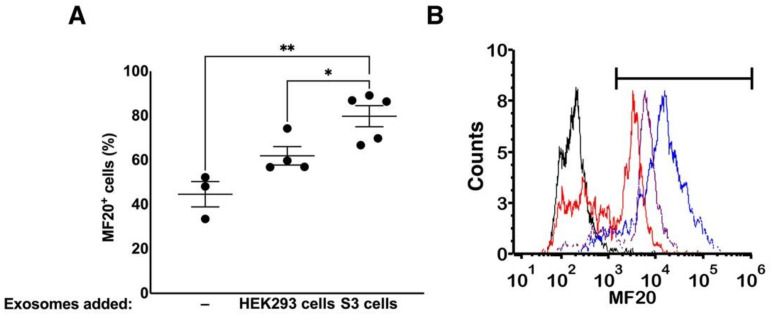
Effect of exosomes on differentiating hPSCs. (**A**) Exosomes from S3 B12-3 cells (50 µg) were added to S2 cultured B12-3 cells (confluent wells of a 12-well plate) inducing an increase in MF20^+^ cells at day 8 as mean ± SEM; * *p* = 0.0457, ** *p* = 0.016. (**B**) A representative flow cytometry run from the experiments in (**A**) is shown. Curves: Black-Isotype control; Red-no added exosomes; Purple-HEK293 cell exosomes added; Blue-S3 exosomes added.

Flow cytometry data taken at day 8 of differentiation (Figure 7A,B) revealed that addition of exosomes had a positive effect on differentiation as the fraction of MF20^+^ (MYH1E) cells [31,32,33] increased from 44.63 ± 9.86% (without additional exosomes) to 79.77 ± 10.61% (*p* = 3.53 × 10^−3^) for cells cultured with exogenous S3 exosomes. In contrast, the addition of exosomes from HEK293 cells did not lead to a statistically significant increase in MF20^+^ cells (61.93 ± 8.67%, *p* = 5.3 × 10^−2^) compared to that of cells cultured without the addition of exosomes. Again, the introduction of S3-exosomes induced a higher specification efficiency vs. that of cells incubated with HEK293 cell-exosomes. These findings point to a potential function of the exosomal cargo on hPSC differentiation and warrant further studies.

## 4. Discussion

Exosomes are emerging as important means of intercellular communication but their cargo and role(s) in hPSC differentiation remain unclear. Here, we focused on examining the protein content of exosomes produced by hPSCs undergoing cardiogenic specification. Pluripotent hPSCs secreted exosomes displaying appropriate characteristics such as relevant markers (CD63, LAMP1, and LAMP2) and size distribution. We used the same isolation method to obtain exosomes from (H9 and B12-3) pluripotent cells and their differentiated progeny. Proteomic analysis performed on exosomes from various differentiation stages revealed the presence of signaling moieties and markers such as NPM1 and CLU at S0, AFM (a Wnt carrier) at S1, PPP2CA (a Wnt signaling promoter) at S1 (H9 cells) and S2 (B12-3 cells), and cardiac cell-relevant proteins such as MYL12A, LDHB in the H9 cell- and ACTC1, WDR1 in B12-3 cell-derived CMs (S3). Exosomes also promoted the differentiation of hPSCs and their progeny opening interesting prospects for stem cell engineering.

Although we detected CD63 by Western blotting in exosomes, proteomic analysis did not yield CD63, LAMP1 and LAMP2 protein signatures. This is expected because these are transmembrane proteins, which are likely degraded during sample preparation for peptide analysis as reported [34]. Low abundance of these proteins also cannot be ruled out since the enrichment of exosomes with various proteins depends on the cell type and culture conditions. We believe that the exosomes were secreted by the ESCRT complex due to the identification of signature markers in our exosome samples [35]. Signature exosomal markers such as CD9 in B12-3 cell-derived CMs, and CD81 and TSG101 in H9 cell-derived CMs were also present. Stage 1 (B12-3) and S2 (H9) exosomes contained ALIX whereas CD81 was detected in exosomal samples from S0 and S2 of B12-3 cell cultures. Of note, proteins putatively interacting with major histocompatibility complex (MHC) II are closely associated with exosomes [36]. Here, HSPA8 and HSP90AB1 [34] were identified in all samples, in addition to YWHAE and RAB7A [34]. Moreover, exosome-related annexin proteins such as ANXA2 [37] (B12-3 cells: all stages; H9 cells: S1), ANXA6 [38] (B12-3 cells: S0, S2, S3; H9 cells: S1), MFGE8 (B12-3 cells: S0) [39] and ANXA5 [40] (B12-3 cells: S0, S1, S3; H9 cells: S1, S2, S3) were observed. These proteins localize in the endosome or are associated with multivesicular body loading.

A strong correlation was noted between almost all pairs of our datasets based on PCC values, except for B12-3 cell exosomes in S2 and S3. The correlation was performed using only those proteins that had a non-zero normalized intensity in both the datasets being compared. Therefore, the comparison included largely constitutive, translational, or metabolic proteins. The high correlation between the first and third stages for H9 cells can be attributed to the fact that S3 exosomes had many proteins—especially proliferation-related ones—in common with S1. While investigating the reason for such differences, we observed that B12-3 S1 and S2 generally presented higher and more varying levels of proteins when compared to S0 and S3 of both lines. S0 exosomes carried relatively high levels of extracellular proteins such as HP, A1BG, HPX, HBB, and AFM. Exosomes secreted by hPSC-derived CMs (S3) carried the highest amount of exosome-associated proteins (SDCBP/syntenin, CLTC), metabolic proteins that are abundant in cells with high metabolic activity (CKB [41], PKM [42]), and CM-enriched proteins (ACTC1 [43], SERPINE2 [44]). Multifunctional proteins were also detected: HNRNPU, which is required for mitosis, POU5F1 (OCT4) regulation in stem cells [45], and cardiac development [46]; UBA1, which is linked to protein homeostasis, stabilization of short-lived proteins, and temperature-sensitive cell cycle arrest [47]; RHOA (RhoA GTPase), which promotes Wnt activity in proliferating cells [48], and it is a downstream product of the non-canonical TGF-β pathway, ensuring cell adhesion and cytoskeletal reorganization, in conjunction with CDC42 [49,50].

Stage 3 exosomes had the highest levels of proteins which are involved in cardiogenic processes. Our analysis of the datasets using PCA demonstrated a less marked divide of the datasets by stage of differentiation in PC3. The majority of the H9 sets were clustered near the zero line because there were fewer unique proteins in each H9 cell dataset, and their values were closer to the average. On the other hand, two datasets (S2 and S3) were unique in the B12-3 line. S2 exosomes in the B12-3 line contained a high number of unique proteins, separating it along PC1. This included proteins that influence differentiation such as IGF2BP1, TRA2B, PSMC3, PSMC5, and PSMC6, while S3 for H9 cells contains a significant enough number of proteins with values deviating from the averages to be separated along PC2 and PC3.

Exosomes isolated from pluripotent stem cells (S0) contain proteins which prevent differentiation and promote self-renewal including ‘stemness’ markers such as OCT4, NANOG, SOX2, as well as proteins involved in stabilizing these markers (SSB [51,52], RPS24 [51,52,53], RPL7A [54], RPL14 [55], RPL24 [51,55], RPL6 [51,53], RPL9 [56], CRABP1 [51], EIF4A1, SERPINH [52,55]). The expression of RPL7A and RPL9 is high in pluripotent stem cells and is reduced as cells differentiate [56]. We detected RPL7A in all datasets with the greatest level in S1 (H9 cells) and S2 (B12-3 cells). RPL9 is observed in exosomes from self-renewing cells for both hPSC lines peaking at S2 and S3 (B12-3 cells). Proteins such as SFPQ [57] and KPNA2 [58] are responsible for maintaining pluripotency. NPM1 is responsible for proliferation and cell fate determination [59]. EIF4A1, present in all datasets but H9 S3, is involved in cell proliferation [60]. The only metabolic proteins specific to exosomes from the pluripotency stage of both lines are APOA2 (lipid metabolism and transport) and ATP5B (ATP synthesis). Certain proteins that are associated with the upkeep of pluripotency are present only in B12-3 S2 exosomes (RPS5 [51], SRSF3 [61], and TRIM28 [62,63]), while RPS20, which is expressed preferentially in hPSCs than differentiated cells [56] was detected only in exosomes from S1 and S2 of B12-3 cells. Overall, B12-3 S2 exosomes had a relatively high number of pluripotency genes even surpassing S0 exosomes from both lines. This raises the possibility that cells undergoing specification clear pluripotency-maintaining moieties via exosomes.

Notably, the H1F0 protein that is present in low proliferative capacity cells and is involved in apoptosis of proliferating cells [64], was observed in exosomes from S2 of the H9 line. H1F0 is also detected in cancer cell exosomes and a parallel mode of action may be applicable in differentiating stem cells.

Activation of the canonical Wnt and TGF-β signaling pathways in hPSCs led to the emergence of T^+^ mesendoderm cells (S1). Exosomes from both hPSC lines at S0 and S1 contained the Wnt carrier AFM [65]. Another Wnt enhancer [66,67], PPP2CA, was detected in exosomes from H9 cell-derived T^+^ cells S2 B12-3 differentiated cells. The mesendoderm layer arises through the primitive streak formation in vivo with the participation of non-muscle myosin heavy chains II a and b (MYH9, MYH10) [68]. These myosins are activated via Rho-kinase signaling. We observed the presence of MYH9 in exosomes from S1 and S2 (H9 cells) and S2 and S3 (B12-3 cells). MYH10 was detected in S1 (H9 cells) and S0 (B12-3 cells) pointing to interline differences. Myosins are expressed throughout development to various extents, therefore, their presence in exosomes from different stages is not unexpected. Other late stage mesoderm markers such as PODXL (a cardiac progenitor marker [69]) were noted in exosomes from S1 of H9 cell cultures.

Two days into the differentiation, exogenous Wnt inhibition was applied resulting in the emergence of cardiac mesoderm (S2) cells displaying KDR, PDGFRA, and NXK2.5. The respective exosome datasets (B12-3) contained two splicing proteins, HNRNPU [46] and TRA2B [70], which when knocked down, cardiogenesis is inhibited. MYL12A, which is expressed in the sarcomere structure of ventricles [71] and is required for cardiac progenitor formation [72], was identified in S2 exosomes from B12-3 cell cultures. Although we did not observe proteins directly involved in intrinsic Wnt signaling inhibition, PSM complex proteins (PSMC3, PSMC5 [73], PSMC6) were present for the first time in S2 exosomes (B12-3). These proteins mediate the degradation of β-catenin [74], with PSMC5 specifically binding to ubiquitinated Wnt ligands. The ubiquitin enzyme that targets β-catenin, UBA1 [75], has also been observed in the same dataset. Although this does not provide evidence of any Wnt inhibition mechanism, it supports the potential role of S2 exosomes in transferring species targeting intracellular β-catenin.

Also detected in exosomes in this stage is IGF2BP1, which inhibits IGF2 attenuating the effects of insulin [76]. We have not modulated the IGF pathway externally, therefore the presence of a protein from this pathway warrants further investigation.

On continued Wnt inhibition until day 8, progeny emerged expressing TNNT2 (>85% by day 12). Isolated exosomes carried moieties suggesting continued Wnt inhibition. Detected GPC4 is responsible for cardiac development via BMP and Wnt signaling attenuation [77]. Another protein involved in TGF-β inhibition in both lines is HSPA1A [78]. Cardiac-specific proteins were detected in S3 exosomes from both hPSC lines. H9-derived CM exosomes contain metabolism-associated molecules such as the ATP-hydrolyzing ATP1A1 (for electrochemical Na^+^/K^+^ gradient and contractile activity [79]) and lactate-forming LDHB (relevant to cardiac muscles and abundant in ventricles [71,80]). YWHAE is implicated in several signaling cascades including those responsible for regulating cardiac rhythm via K^+^ and Ca^2+^ channels [81,82]. CTNNA1, which promotes CM maturation and hinders proliferation [83,84], WDR1, which participates in ventricular development and sarcomere formation [85,86], and EDH1, which facilitates contractile activity and connexin junction formation [87,88,89], were among the proteins detected in exosomes from CMs in B12-3 cell cultures.

A shift in metabolism is well documented for differentiating stem cells from glycolysis in the pluripotent state to oxidative phosphorylation (OxPhos) [90,91]. Accordingly, S0 and S1 rely on glycolysis and—to a lesser extent—glutamine oxidation for metabolism. Glycolysis in self-renewing and differentiating hPSCs [92] is aided by the glucose transporter GLUT1 (SLC2A1). GLUT1 was detected in exosomes from S1, S2, (H9/B12-3 cells) and S3 (B12-3 cells). GLUT3 (SLC2A3) [92] was also contained in exosomes from S1 (B12-3 cells) and S2 (H9 cells). Oxidation of pyruvate and lactate becomes more pronounced during S2 (cardiac mesoderm) and S3 (cardiac progenitors). To this end, the glycolytic enzymes ENO1, PKM, and GAPDH were carried by exosomes at all stages of specification for both lines. ALDOA, a muscle-specific glycolytic enzyme, and PFKP were identified in exosomes from different stages. PHGDH, an enzyme involved in OxPhos [93] was identified in the proteomic profiles of exosomes from various stages with the lowest levels noted in pluripotent cell vesicles and increasing with differentiation. Immature CMs (S3) employ lactate oxidation as their main energy source, followed by FA metabolism for mature CMs. LDHA was detected in S1, and cardiac-specific LDHB in H9 cell-derived CMs. The FA transporter protein APOE was detected in S3 exosomes. Moreover, the presence of APOE, APOA2, or FA transporters in S0 exosomes may be attributed to the importance of lipid synthesis in proliferating pluripotent stem cells [94,95].

Exosomes from H9 hESCs differed from those isolated from B12-3 hiPSCs in the number of proteins detected. B12-3 cell exosomes contained more proteins in S0, S2, and S3 whereas H9 cell exosomes had a larger number of proteins in S1. Additionally, exosomes for both lines at each stage contained proteins involved in proliferation, metabolic, translational, and exosome-associated processes. Signaling proteins detected in exosomes from both lines include insulin and Wnt-associated proteins (AHSG, AFM) at S0 and S1. However, stage-specific proteins at S1 are present in the H9 hESC exosomes rather than in the ones from B12-3 hiPSCs. This may indicate a higher mesoderm differentiation efficiency for hESCs. As noted in the PCA, S2 exosomes from differentiating hiPSCs stand out. This translates to several stage-appropriate proteins being present (e.g., TRA2B, HNRNPU). In contrast, there is a conspicuous absence of stage-specific proteins in the H9 cell exosomes at the same stage.

Although structural and metabolic proteins relevant to cardiac cells are recorded in exosomes from both lines, they do not overlap. In S3, exosomes from both hPSC lines contain SERPINE2, an abundant protein in the heart [44], and HSPA1A, which in addition to being an exosome-associated protein, mediates Smad3 degradation curtailing the effects of TGFβ signaling [78]. Yet, G-proteins are found in H9-derived CM exosomes but not in those from B12-3 CMs. Similarly, GPC4, which is a Wnt and BMP4 signaling inhibitor [77], was detected only in H9 CM exosomes, even after exogenous inhibition of Wnt ceased. In turn, CTNNA1, was observed in B12-3-CM exosomes but not in those from H9-derived CMs. These differences in signaling intermediates in the exosomal cargo of the hESC and hiPSC lines employed here may reflect analogous cytoplasmic differences and thus altered signaling states during cardiogenic specification. Such variations in intracellular signaling among hPSC lines have been reported and corroborated by the disparate concentrations of soluble cues required to achieve similar differentiation efficiencies [96,97].

The existence of proteins, which are linked to differentiation, within exosomes from hPSC cell-derived CMs motivated the utilization of those exosomes to determine their effect on the specification process. HEK293 cell-derived exosomes contain proteins promoting the formation of CMs indirectly such as insulin-binding and -degrading factors, TPM4, WDR1, α-catenin, TNC, and EHD1. This may contribute to the observed trend of increased differentiation efficiency with the addition of exosomes isolated from HEK293 cells, but this was not significantly different from control samples with no added exosomes.

The addition of S3 exosomes to hPSCs undergoing commitment led to a definite improvement in efficiency. While this effect may not be solely due to the protein content of exosomes, but to other cargo elements (e.g., mRNA, miRNA) as well, the net enhancement in differentiation efficiency merits further study. The proteomic cargo of exosomes produced by hPSCs differentiating toward CMs can be mined more extensively to identify line- and stage-specific moieties which collectively regulate commitment.

## 5. Conclusions

Our results point to a role of exosomes in enhancing cardiogenic differentiation and open avenues to explore similar functional modes in hPSC commitment to other therapeutically important lineages. The analysis has uncovered several proteins which are involved in cardiopoiesis-related processes. Through further mining of the proteomic data from this study, deeper insight can be gained about the molecular mechanisms mediated by exosomes and candidates can be identified for enhancing the differentiation and maturation of hPSC-derived CMs, facilitating the development of cellular therapies for heart disease.

## Data Availability

Proteomic data generated in this study have been deposited to the PRIDE database (https://www.ebi.ac.uk/pride/) with accession number PXD027888.

## Supplementary Materials

The following are available online at https://www.mdpi.com/article/10.3390/cells10102622/s1, Figure S1: Whole blots for GRP94, CD63, LAMP1 and LAMP2 corresponding to the images shown in Figure 2C, Figure S2: Relative miRNA expression of HEK293 cells incubated with 100 μg of exosomes produced by H9 hESCs, Table S1: Primers used in this study, Table S2: Common proteins among different data sets, Table S3: Common proteins among all data sets (see Table S2).
