# Supplementary material for: Proteomic Analysis of Exosomes during Cardiogenic Differentiation of Human Pluripotent Stem Cells
Source: Cells. 2021 Oct 1;10(10):2622. doi: 10.3390/cells10102622 (PMC8533815; doi:10.3390/cells10102622)
Supplement: Supplementary file 1 [file cells-10-02622-s001.zip › cells-1396530-supplementary.pdf]

__Supplemental Figures__

Proteomic Analysis Of Exosomes During Cardiogenic Differentiation Of Human Pluripotent Stem Cells

Preeti Ashok, and E. S. Tzanakakis

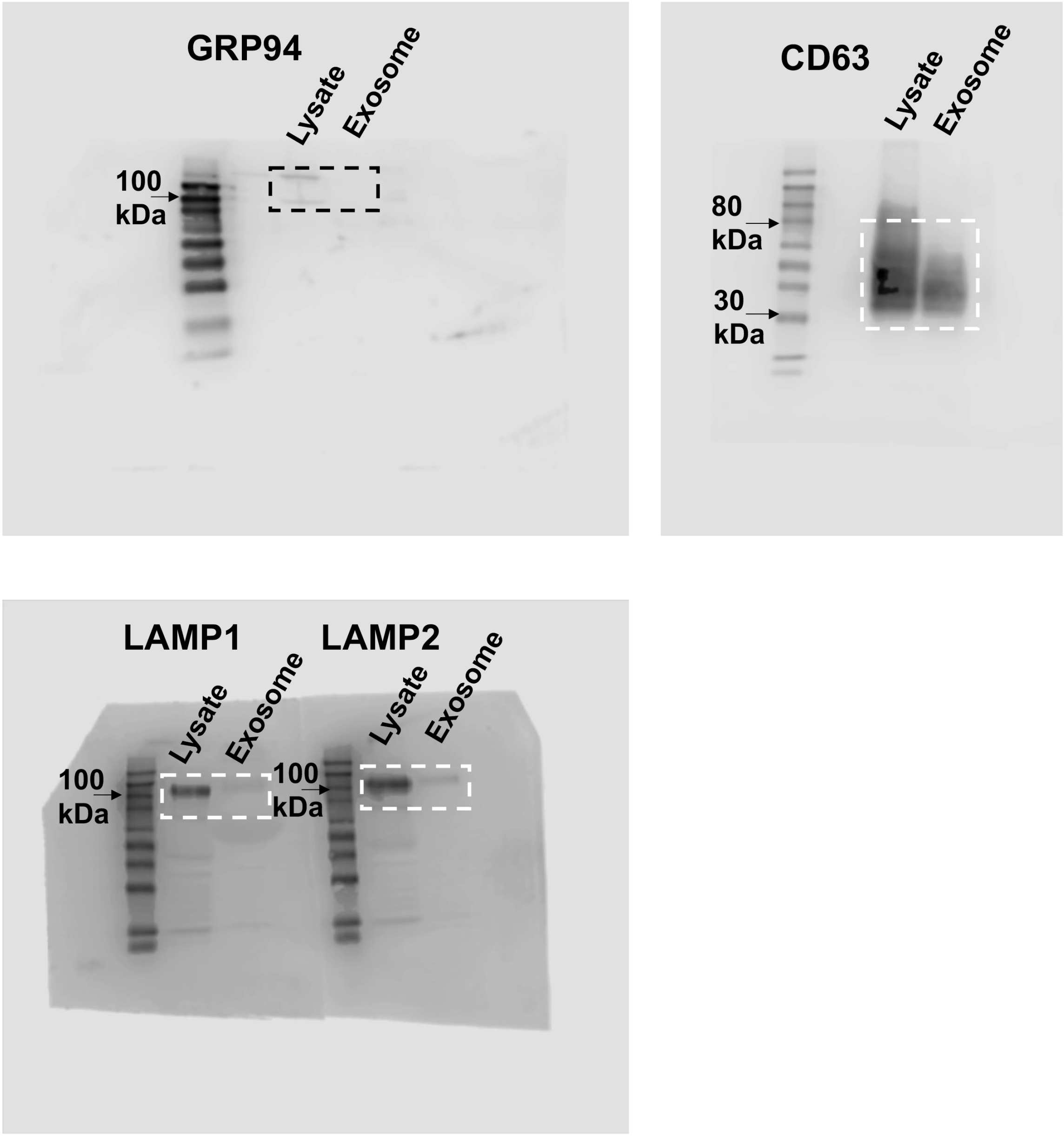

Figure S1. Whole blots for GRP94, CD63, LAMP1 and LAMP2 corresponding to the images shown in **Figure 2C**. Detected proteins are indicated with boxes.

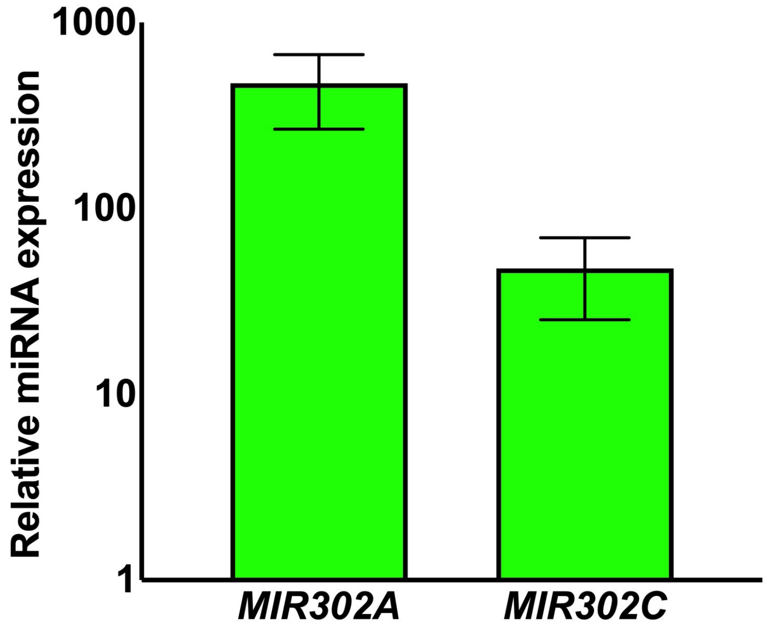

Figure S2. Relative miRNA expression of HEK293 cells incubated with 100 μg of exosomes produced by H9 hESCs. Results are shown as mean ± SD from a triplicate experiment.
